# An investigation of PCT and EU patents on helmets to improve thermal comfort

**DOI:** 10.1186/2046-7648-4-S1-A88

**Published:** 2015-09-14

**Authors:** JuYoun Kwon

**Affiliations:** 1 School of Design and Human Engineering, Ulsan National Institute of Science and Technology, South Korea

## Introduction

To develop a product for people who are exposed to the particular environment or activity, their implicit need should be determined; their behaviour, activity, and task etc. can be investigated. A survey was conducted to investigate the implicit need of boating people [1]. 32.1% of respondents thought that 'head' is the most vulnerable body part to get injured while boating and 11.7% of the respondents had been injured on the 'head' indicating boating people require safety equipment to provide a protection of their head. Therefore, the aim of this study was to investigate the technology used for developing helmets through analysing the helmet patents of Patent Cooperation Treaty (PCT) and EU.

## Methods

The WIPSON database [2] was used in order to analyse the patents of PCT and EU and the search keyword was helmet. The total numbers of PCT and EU registered or publicised were 412 cases and 428 cases respectively from January, 1^st^, 2011 to December, 31^st^, 2014. The patents to improve thermal comfort for wearers were distinguished after considering abstract, exemplary claim, plans and International Patent Classification (IPC). IPC is made up of eight sections; A: 'Human necessities', B: 'Performing operations; transporting', C: 'Chemistry', D: 'Textiles', E: 'Fixed constructions', F: 'Mechanical engineering; lighting; heating; weapons; blasting', G: 'Physics', H: 'Electricity'. The section was subdivided into further four sublevels of 'class, subclass, group, and subgroup' [3]. The IPC's descriptions on the levels of 'section and subgroup' were considered to investigate the technology used for developing helmet.

## Results

The common contents of both PCT's and EU's were about the technology related to respiratory, moulding & shaping, layered products composed of specific materials, safety technology adapted for vehicle, attaching technology, shock-absorbing technology, lighting, optical parts or element, signalling, and transmission/transducer technology. The number of PCT patents for improving thermal comfort was 14 cases and the number of EU patents was 13 cases. These patents were classified into at least one of eight sections in IPC. 12 cases of PCT patents and 11 cases of EU patents belonged in section A only. A patent of PCT and a patent of EU were affiliated to section B as well as section A. A patent of PCT belonged in both sections B and F. A patent of EU was affiliated with both section A and section F. Additionally, of the subgroups considered, the patents relating to improving thermal comfort were classified into six kinds of subgroups in maximum. PCT patents tended to belong to 'Parts, details of accessories of helmets', 'Ventilation arrangement', and 'Filtering process or with filter elements' but most of EU patents belonged to 'Ventilation arrangement'. However, there were few patents in which sensor system was used but the PCT patent and the EU patents belonged to 'Electric communication technique' and 'Parts, details or accessories of helmets'/'Cushioning device' respectively.

## Conclusions

The characteristics of PCT patents were about supplying additional parts of a helmet such as a mask of helmet with filters, a cooling/ventilating fan, shutters for ventilation, an absorber for CO_2_, a compact air purifier, and a liner of breathable fabric etc. but the contents of EU patents tended to be about modifying the combination of shells or foam or transforming the original shape of shells or foam.

## References

[B1] Kwon J Kyung G A survey on Sailing-related Injuries and Usage of Personal Protective Equipment (PPE) Proceedings of 6th European Conference on Protective Clothing 2014 Belgium May 2014

[B2] WIPSON http://www.wipson.com

[B3] WIPO http://www.wipo.int/classifications/ipc/en/

